# Supplementary material for: Large-scale DNA-based phenotypic recording and deep learning enable highly accurate sequence-function mapping
Source: Nat Commun. 2020 Jul 15;11:3551. doi: 10.1038/s41467-020-17222-4 (PMC7363850; doi:10.1038/s41467-020-17222-4)
Supplement: Supplementary file 1 — Peer Review File [file 41467_2020_17222_MOESM1_ESM.pdf]

Reviewers' Comments:

Reviewer #1:
Remarks to the Author:
The authors propose a biotech method called uASPIRE. It uses a short DNA containing a gene, a GRE and a discriminator. When the GRE increases the expression of the gene, the discriminator gets altered. By putting a lot of short DNAs containing various GREs into Ecoli and reading out the fraction of discriminators altered, we get a profile of GREs about their effect. In an experiment, they obtained the activities of 303503 RBSs over 9 time points. I think it is a useful technique, but I have several reservations.

1. Is it appropriate to say that the method is doing phenotypic profiling? uASPIRE measures the activity of gene regulatory elements to a specific gene. I think the GRE activity is not called "Phenotype" in general.

2. In Introduction, the authors claim that it is free from bias unlike other methods, but the measurements of uASPIRE is also done in a very artificial system on a short DNA sequence. So it might be different from the GRE activity on a real genome. How do you evaluate the bias from this aspect?

3. Related to above, all the results presented are coming from uASPIRE and there were no comparison to another method. If the authors can compare uASPIRE with other techniques in at least one simple experiment, their argument would be more powerful.

4. The experiments in ecoli are interesting, but probably most biologists are interested in other model organisms and human. Can this method be applied to other organisms?

5. The machine learning model tries to predict the IFP (integral of the flipped profile) that essentially measures the GRE activity. Interestingly, it predicts a distribution of IFP instead of a value of IFP. Can we understand it as maximum likelihood estimation or Bayesian optimization? What is the statistical rationale behind the estimation of the distribution.

6. The evolution experiments in Fig 5(g),(h) are interesting. Oddly, it evolves from the sequence with smallest IFP towards larger IFP, and vice versa. In biotechnological application, one may need a sequence whose IFP is higher than the highest IFP observed so far. If the authors can design a new sequence with very high predicted IFP and validate it with experiments, this is the sign that the machine learning model is really working.

Reviewer #2:
Remarks to the Author:
The authors developed a method that relies on DNA based phenotypic recording and deep sequencing to record more than 2.7 million sequence-function data points in a single experiment to kinetically measure 75 translation from 303,503 RBS variants in Escherichia coli. I like the idea but the authors should cite a previous paper using the DAM methylase and deep sequencing by Yus et al in Nature comm which follows the same spirit (Yus E, Yang JS, Sogues A, Serrano L. A reporter system coupled with high-throughput sequencing unveils key bacterial transcription and translation determinants. Nat Commun. 2017 Aug 28;8(1):368. DOI: 10.1038/s41467-017-00239-7). Although the genes used are not the same the philosophy and idea behind is the same. They described a three-component DNA architecture comprising a genetic
sequence to be investigated ("diversifier"), the gene of a DNA-modifying enzyme ("modifier"), and the cognate DNA substrate of this enzyme ("discriminator"), all located on the same DNA molecule. In Yus et al., you have exactly the same composition
GTAC sequence to be methylated ('cognate sequence), a gene sequence to be investigated

('diverisfier') and the DAM methylase ('modifier'). The advanatge of the DAM is that by having several GTAC sequences in one single bacteria one can get a quantitative stimate of the amount of DAM being produced, while in this paper is a boolean decision for a single bacteria. An advantage of their method is that they do not need to digest the DNA with two different enzymes prior to sequencing. The big disadvantage of the authors method is the tight regulation of the expression of their recombinase.
Aside from that they did an elegant study, with an interesting analysis of the sequence data through the SAPIEN tool they developed.

Reviewer #3:
Remarks to the Author:
This manuscript describes an innovative approach to mapping the effect of gene regulatory elements, in their case ribosome binding sites (RBS). The basic strategy, which involves an irreversible recombinase, bypasses many of the measurement limitations associated with using large sequence libraries. Their key innovation is that the response is sequenced based, which enables large-scale characterization of the RBS library.

Overall, this is an impressive and comprehensive piece of work. My only concern is that the analysis part seems somewhat limited. In particular, can you derive some more substantive conclusions from the data and machine learning. One suggestion would be see whether the neural network could be used to design RBS's with known activity. However, given the richness of the dataset, this seems to be a mundane task. Another suggestion would be to apply an adversarial approach to identify single mutations that take a weak sequence and make it a strong one. Such an exercise, though not necessary, would greatly strengthen the manuscript.

Minor points:

1. How much overlap was there between the testing and training sets? Can you provide an example where the neural network failed?

2. Line 348: I was surprised that there was no correlation between the folding energy and activity. Did any of the sequences form stable hairpin? If so, can you elaborate?

3. In Figures 5g and h, did you actually test these sequences? For example, the strong one does not match the canonical SD sequence.

4. The ML annex is a nice addition. It is very readable.

5. How much does context matter? If you used these RBS's to drive the translation of a different protein on a different plasmid (or chromosome), would the neural network still work? Clearly, addressing this question is beyond the scope of this work but it is something to consider and perhaps address. My personal and perhaps biased experience is that only the strong and weak sequences translate well to other contexts. The intermediate strength sequences are the problematic ones.

Dear editor, Dear reviewers,

We cordially thank the reviewers for their efforts, positive feedback and the valuable input on our manuscript. Hereafter, we provide a point-by-point reply (in blue) to the individual points raised by the reviewers. Corresponding changes to the manuscript are highlighted in tracked changes in the respective docx files.

Best regards,

The authors

****

Reviewer #1 (Remarks to the Author)

The authors propose a biotech method called uASPIRE. It uses a short DNA containing a gene, a GRE and a discriminator. When the GRE increases the expression of the gene, the discriminator gets altered. By putting a lot of short DNAs containing various GREs into Ecoli and reading out the fraction of discriminators altered, we get a profile of GREs about their effect. In an experiment, they obtained the activities of 303503 RBSs over 9 time points. I think it is a useful technique, but I have several reservations.

1. Is it appropriate to say that the method is doing phenotypic profiling? uASPIRE measures the activity of gene regulatory elements to a specific gene. I think the GRE activity is not called "Phenotype" in general.

The authors thank this reviewer for this comment. We argue that term “phenotypic” refers to any observable, functional characteristic that an organism exhibits as a result of genetic features of its genome. To this end, different phenotypes may well arise due to differential gene expression (in our case via different GREs in otherwise isogenic populations). Further, we argue that the method is not restricted to GREs *per se* and could, for instance, be used to assess different recombinase variants with minor adaptions. We do however appreciate that the term “phenotype” is very differently interpreted depending on the field. In order to avoid potential confusion we added the following sentence to the manuscript: “We define the term “phenotypic” as any observable, functional characteristic that arises from a genetic sequence” (lines 97-98).

In Introduction, the authors claim that it is free from bias unlike other methods, but the measurements of uASPIRE is also done in a very artificial system on a short DNA sequence. So it might be different from the GRE activity on a real genome. How do you evaluate the bias from this aspect?

The authors would like to clarify that there was no intention to claim that the method is absolutely free from bias, but to point to different characteristics of uASPIre that allow to reduce or avoid specific known sources of technical bias that occur in alternative methods, for example PCR amplification and barcoding bias, and avoidance of bias due to differential processing times in sequential variant analysis. We believe that the initially used phrase “eliminates bias” in the introduction could be misinterpreted and therefore replaced it with “eliminates technical bias associated with sample processing” (lines 72-73).

Indeed, absolute expression levels will be different in the case of chromosomal integration since gene copy number (amongst numerous other things) is a known influencing factor. Importantly, we determine/predict relative GRE activities that allow comparison of different GREs in the same context, which should (with certain limitations) be valid also for the case genomic integration. Further, we would like to point out that overexpression of proteins in E. coli and other microorganisms is indeed performed on the basis of plasmids with multiple copies and not by genomic integration.

2. Related to above, all the results presented are coming from uASPIRE and there were no comparison to another method. If the authors can compare uASPIRE with other techniques in at least one simple experiment, their argument would be more powerful

We agree with the reviewer on the importance of comparisons to other methods, which we indeed already do by comparing the uASPIre readout with GFP measurements (as for instance used in Flowseq). This comparison was performed for the 31 internal-standard RBSs in triplicate shake flask cultivations (compare Fig. 3d and Suppl. Fig. 11). The experiments indicated an improved sensitivity and a larger dynamic range of the NGS readout compared to the fluorescence measurements (see lines 268-270 in the manuscript).

3. The experiments in ecoli are interesting, but probably most biologists are interested in other model organisms and human. Can this method be applied to other organisms?

While this proof-of-concept study was performed in bacteria, we would like to emphasize that in principle the method is by no means restricted to prokaryotic systems. We argue that it can be applied to other organisms as long as monoclonality can be preserved and a suitable DNA-modifying enzyme can be used in the desired host (note that Bxb1 has been shown to be active also in eukaryotes and even human cells; alternative recombinases are readily available). We added the following sentence to the discussion: "Lastly, we expect uASPIre to be applicable in a wide range of host organisms, since Bxb1 is functional in pro- and eukaryotes including human cells[33,34] and a variety of well-characterized alternative recombinases and other DNA-modifying enzymes are readily available."(lines 483-486).

4. The machine learning model tries to predict the IFP (integral of the flipped profile) that essentially measures the GRE activity. Interestingly, it predicts a distribution of IFP instead of a value of IFP. Can we understand it as maximum likelihood estimation or Bayesian optimization? What is the statistical rationale behind the estimation of the distribution.

The main motivation behind this choice was to model a part of the predictive uncertainty, here the aleatoric uncertainty. Aleatoric uncertainty typically accounts for the stochasticity of the data that is intrinsic to the data generation process, e.g. coming from measurements or noise. In this way, estimating a predictive distribution allows us to characterize both the expected value of the IFP as well as its spread (uncertainty), which we quantify as the standard deviation of the predictive distribution. Crucially, such a sequence-by-sequence characterization of predictive uncertainty would not be feasible with a model that only provides pointwise predictions. We clarified the rationale behind the estimation of the distribution (lines 298-302).
Our approach employs maximum likelihood estimation rather than a Bayesian technique.
In more detail:

- We employ maximum likelihood estimation (MLE) to fit the parameters of the predictive distribution to the data (or, more precisely, to fit the weights of the neural network layers that output the parameters of the predictive distribution as a function of the input sequence). To this end, by using the negative log-likelihood (NLL) as our loss, the model is incentivized to capture both the location and spread of the predictive distribution, as opposed to pointwise losses such as the mean-squared error (MSE), which would be concerned only with the location.
- This is an effective way to model aleatoric uncertainty, and is not directly related to Bayesian techniques. In contrast, accounting for epistemic uncertainty (i.e. uncertainty caused by our ignorance about the correct model parameters) typically requires a fully-Bayesian treatment of the problem, which remains an open problem for deep learning models such as SAPIENs. A simple yet surprisingly effective and widespread approach to tackle this problem, which we adopted as part of SAPIENs, is to fit an ensemble of models, with each model in the ensemble trained

independently of the others from a different random initialization and ordering of minibatches (Lakshminarayanan et al., Adv. in Neural Information Processing Systems 30, 6402-6413 (2017)). Intuitively, this allows to estimate the epistemic uncertainty by quantifying to which extent the different elements of the ensemble agree in their predictions. More details about the relationship between parameter estimation and ensembling are given in the ML Annex Section A11.

- Importantly, we would like to emphasize that the combination of both approaches appears to succeed in capturing the main sources of uncertainty, as confidence intervals of any width are well calibrated as we show in Figure 4f.

5. The evolution experiments in Fig 5(g),(h) are interesting. Oddly, it evolves from the sequence with smallest IFP towards larger IFP, and vice versa. In biotechnological application, one may need a sequence whose IFP is higher than the highest IFP observed so far. If the authors can design a new sequence with very high predicted IFP and validate it with experiments, this is the sign that the machine learning model is really working.

We thank Reviewer 1 for this comment, which raises an important question regarding the validation of the model. We believe that the following two observations in our manuscript demonstrate the ability of the machine learning model to accurately predict unseen (and in particular strong) sequences.
First, we were able to experimentally validate sequences that were generated by a simplified version of SAPIENs with the objective to obtain intermediate-to-strong RBS sequences, by training on the small dataset (~10,000 samples; ML Annex Section B3). These RBSs indeed showed intermediate to strong activity (library “High3” in Fig. 3e, compare Suppl. Fig. 7a).
Second, we would like to point out that the IFP takes values between 0 and 1, with a value of 1 corresponding to a hypothetical instantaneous flipping of all discriminators at the time point of induction. Therefore, experimentally designing sequences that have IFPs larger than 1 is not possible. In our dataset, we find many RBSs with IFPs greater than 0.8, which indicates that these are already extremely strong sequences. Additionally, some of the internal-standard RBSs were designed to be very strong using the “RBS calculator” (Howard Salis, Penn State), a benchmark prediction algorithm for RBSs. Strong activity of these sequences was also confirmed by GFP measurements, which reached the level of saturation for the strongest RBSs as shown Figure 3d. Importantly, SAPIENs predicts the behavior of such strong sequences, which were excluded from training, with high accuracies (see for instance the rightmost violins of Fig. 4c and the rightmost points in Fig. 4d).

Reviewer #2 (Remarks to the Author)

The authors developed a method that relies on DNA based phenotypic recording and deep sequencing to record more than 2.7 million sequence-function data points in a single experiment to kinetically measure 75 translation from 303,503 RBS variants in Escherichia coli. I like the idea but the authors should cite a previous paper using the DAM methylase and deep sequencing by Yus et al in Nature comm which follows the same spirit (Yus E, Yang JS, Sogues A, Serrano L. A reporter system coupled with high-throughput sequencing unveils key bacterial transcription and translation determinants. Nat Commun. 2017 Aug 28;8(1):368. DOI: 10.1038/s41467-017-00239-7). Although the genes used are not the same the philosophy and idea behind is the same. They described a three-component DNA architecture comprising a genetic sequence to be investigated (“diversifier”), the gene of a DNA-modifying enzyme (“modifier”), and the cognate DNA substrate of this enzyme (“discriminator”), all located on the same DNA molecule. In Yus et al., you have exactly the same composition: GTAC sequence to be methylated ('cognate sequence), a gene sequence to be investigated ('diverisfier') and the DAM methylase ('modifier'). The advanatge of the DAM is that by

having several GTAC sequences in one single bacteria one can get a quantitative stimate of the amount of DAM being produced, while in this paper is a boolean decision for a single bacteria.

The authors agree on the importance of the article of Yus et. al. and would like to emphasize that this article was actually cited in the manuscript (please see Ref 24). To further highlight the importance of this study we added the following passage to the introduction:" In a particularly noteworthy recent study, Yus and coworkers have used dam methylase as a reporter to facilitate a functional readout that can be quantified by NGS with high throughput[24]." (lines 53-55).

Nevertheless, we are convinced that our approach offers several characteristic benefits compared to available methods. These include the high accuracy/quality and internal normalization of the functional readout and the avoidance of bias from PCR amplification and extensive sample processing procedures. Further, the high orthogonality of the recombinase reporter, which is granted due to its high sequence specificity, is an important aspect. DNA methylation, for instance, does not offer this "orthogonality" since it affects transcription, plasmid copy number and cell cycle control (e.g.: https://doi.org/10.1016/j.mib.2005.02.009), and overexpression of dam methylases is often accompanied by toxic effects (e.g.: https://doi.org/10.1016/j.devcel.2013.05.020). We added a corresponding sentence to the manuscript (lines 120-122).

Importantly, we would like to emphasize that in the case of uASPIre, the determined functional readout is not binary/boolean, not even for a single bacterium. The use of multi-copy vectors and sequencing of several copies of the discriminator per genetic variant facilitate highly quantitative and precise measurements as we amply demonstrate throughout the manuscript (please note that for instance for the data shown in Fig. 2c and 3b, an average of 18,700 and 551 discriminator copies per diversifier variant were read, comprising respectively, ~14 and ~9 bits of information per diversifier variant).

An advantage of their method is that they do not need to digest the DNA with two different enzymes prior to sequencing.

We agree with the point that this reviewer makes and thank her/him for this comment. An important feature of our approach is the avoidance of any differential sample processing and PCR amplification steps (note that we and others have shown that PCR amplification can be a major source of bias).

The big disadvantage of the authors method is the tight regulation of the expression of their recombinase.

We argue that this is a solvable technical issue as we demonstrate in our study by using a rhamnose inducible system. To this end, several other tightly regulated promoter systems have also been introduced, which may likewise be used. An alternative solution would be to implement a reversal factor for recombination, which is for instance readily available in the case of Bxb1 (https://doi.org/10.1093/nar/gkx567), thereby eliminating the requirement for tight regulation altogether.

Aside from that they did an elegant study, with an interesting analysis of the sequence data through the SAPIEN tool they developed.

The authors kindly thank this reviewer for this positive feedback and the valuable comments to improve the manuscript.

Reviewer #3 (Remarks to the Author)

This manuscript describes an innovative approach to mapping the effect of gene regulatory elements, in their case ribosome binding sites (RBS). The basic strategy, which involves an irreversible

recombinase, bypasses many of the measurement limitations associated with using large sequence libraries. Their key innovation is that the response is sequenced based, which enables large-scale characterization of the RBS library.

Overall, this is an impressive and comprehensive piece of work.

The authors thank this reviewer for the positive feedback on this study.

My only concern is that the analysis part seems somewhat limited. In particular, can you derive some more substantive conclusions from the data and machine learning. One suggestion would be see whether the neural network could be used to design RBS's with known activity. However, given the richness of the dataset, this seems to be a mundane task.

We agree with this reviewer about the importance of this point, but argue that precisely this was done in our study:

A first model (a convolutional neural network (CNN)) was trained on the initial smaller dataset (~10,000 RBSs), and used to design sequences of intermediate and strong activity via a genetic algorithm (see ML Annex Section B3). The experimental validation of this exercise is presented Figure 3e in the violin plot for the library High3 that indeed was strongly enriched in strong RBSs compared to the fully random library. This is a strong indication that SAPIENs, which is an improved version of the first CNN on a larger dataset, can be used as a basis for forward design.

Further, SAPIENs is able to accurately predict the strength of unseen sequences (Fig. 4b/c, R^2=0.927), which suggests that it should be possible to design sequences with high accuracy for any range of IFP (using e.g. the aforementioned genetic algorithm described in the ML annex Section B3 or the *in silico* evolution model used in Figures 5g/h and described in the Methods section). The design of novel sequences with a certain activity depends mainly on the predictive performance of the underlying prediction model used to assess candidate sequences.

Finally, we would like to point out that we also confirmed that the model predicts both IFP and GFP values of unseen sequences accurately as shown for the 31 internal-standard RBSs (Fig. 4d).

Another suggestion would be to apply an adversarial approach to identify single mutations that take a weak sequence and make it a strong one. Such an exercise, though not necessary, would greatly strengthen the manuscript.

We agree with reviewer 3 in that investigating how to transform a weak sequence into a strong one is a great way to obtain insight about what the model has learnt. However, it seems that single mutations are insufficient to obtain very strong RBSs from very weak ones. This is to be expected for RBS, since such a "gain of function" requires multiple changes in order to achieve sufficient hybridization with the 16S rRNA, which is a well-known requirement to obtain strong RBSs.

Indeed, we carried out such an approach, whose results are depicted in Figures 5g/h. For example, in Figure 5g, starting with a very weak sequence (first row, IFP=0.0007), we explored iteratively which mutations of maximum two bases make this weak sequence the strongest possible, according to SAPIENs' predictions. The result of this first greedy optimization step can be seen in the second row, which led to a predicted IFP of 0.2079. Iterative rounds of this *in silico* mutagenesis were applied until no further increase in strength is reached (last row, IFP=0.9998). Expectedly, the reverse process from strong to weak sequences ("loss of function"; Fig. 5h) seems to be much easier/quicker. Most notably, these sequence-dependent results agree with the global feature importance presented in Figure 5e, which shows which bases the model uses to differentiate strong from weak sequences. This suggests that the model was able to capture which mutations would be required to create strong/weak sequences.

Minor points:

1. How much overlap was there between the testing and training sets? Can you provide an example where the neural network failed?

In the experiments, the training, validation and test sets do not overlap as the datasets were split randomly into disjoint sets.
More generally, reviewer 3 might be referring to the problem of making accurate predictions for out-of-distribution data, which is known to be particularly challenging for machine learning models. The sequences in our dataset span a large set of sequences mostly obtained via full randomisation of 17 bases. Therefore, the notion of out-of-distribution data is less applicable in our setting as train, validation and test sequences to come from (roughly) the same distribution. However, it is possible that our model predicts less accurately the IFP of sequences that are distantly related to any sequence in the training set. If out-of-distribution performance became a priority, this limitation could be solved via extending SAPIENs by incorporating other machine learning techniques that handle dataset shift such as transfer learning or domain adaptation. While we believe that this is out of the scope of the present manuscript, we included an addition to the discussion to acknowledge this possibility (lines 468-472).

2. Line 348: I was surprised that there was no correlation between the folding energy and activity. Did any of the sequences form stable hairpin? If so, can you elaborate?

We agree with the reviewer that this observation seems surprising. We anticipated that the diversification strategy that we used for this proof of concept renders the appearance of strong secondary structures highly unlikely. While a significant overall correlation between folding energy and RBS strength was not observable, strong secondary structures (free folding energy below roughly -15 kcal per mol) showed a tendency to be weak. We included this observation in the manuscript (lines 354-359) and added corresponding figures to the SI (Suppl. Fig. 17a,b). Since sequences with strong secondary structures were underrepresented, we refrained from a deeper analysis of the effect. However, alternative diversification strategies (e.g. designed sequences synthesized on a microarray) may be used, which favor strong secondary structures, in order further analyze their influence. We added a sentence to the manuscript to acknowledge this possibility (lines 359-361).

3. In Figures 5g and h, did you actually test these sequences? For example, the strong one does not match the canonical SD sequence.

The two specific sequences, that the reviewer mentions, were not individually tested for activity. However, we would like to mention that we performed a much more systematic analysis of prediction accuracy on a) ~30,000 RBSs which were recorded by uASPIre but held out during training and b) 31 standard RBSs, again held out during training and tested both by uASPIre and classical individual GFP measurements (shake flask cultivations in triplicates). In this context, we would like to point out that testing of few (additional) individual sequences is insufficient to attest to a high (or low) prediction accuracy of the algorithm, which is why we resorted to the more global analyses mentioned before.

Regarding the strong sequence, which the reviewer mentions, we agree that it does not match exactly the canonical RBS sequence, but also would like to point out that it seems to be quite close to it (the subsequence AGGGGG has a Hamming distance of 1 (A->G) with the canonical SD subsequence AGGAGG). This could be due the fact that a genetic algorithm was used to obtain this sequence *in silico*, a process that does not necessarily lead to the globally most active sequence.

Further, the “canonical SD sequence” (e.g. AGGAGG(U)) is a consensus of E. coli RBSs, but does not necessarily represent the strongest possible sequence. In fact, we find several highly active sequences in our dataset that do not match the consensus.

4. The ML annex is a nice addition. It is very readable.

The authors thank this reviewer for this positive comment on the provided ML annex.

5. How much does context matter? If you used these RBS's to drive the translation of a different protein on a different plasmid (or chromosome), would the neural network still work? Clearly, addressing this question is beyond the scope of this work but it is something to consider and perhaps address. My personal and perhaps biased experience is that only the strong and weak sequences translate well to other contexts. The intermediate strength sequences are the problematic ones.

We agree with the reviewer's comment on the importance of context for RBS activity, which has been amply shown in previous studies. We added a statement to the discussion to highlight the context dependency of RBSs (lines 466-468).
In general, the more the context influences the IFP values, the less the model would be able to accurately predict the IFP value when being transferred from one context to another. It would still be possible to use the collected dataset together with transfer learning or other techniques. However, we agree that this would be beyond the scope of the current study. As reviewer 3 suggests, we also observed that classifying sequences into weak/strong is substantially easier than producing consistently accurate and well-calibrated predictions across the entire range. Therefore, it is plausible that a model trained for this easier task would be more robust with respect to changes in the context. In line with our response to minor point #1, we included in the discussion a sentence about the need of methods such as transfer learning or domain adaptation to be able to generalise to different contexts (lines 468-472) and a paragraph B5 in the ML annex.

****

Reviewers' Comments:

Reviewer #1:
Remarks to the Author:
The authors addressed all of my comments in a satisfactory way. I recommend publishing this paper.

Reviewer #2:
Remarks to the Author:
I am happy with the response and have no objections to the paper being published

Reviewer #3:
Remarks to the Author:
The authors thoughtfully addressed all of my concerns.

Dear editor, Dear reviewers,

the authors would like to cordially thank the reviewers for their efforts, positive feedback and the valuable input on our manuscript. Hereafter, we provide a point-by-point reply (in blue) to the individual points of the reviewers.

Best regards,

The authors

****

Reviewer #1 (Remarks to the Author):

The authors addressed all of my comments in a satisfactory way. I recommend publishing this paper.

The authors kindly thank this reviewer for his support and positive recommendation.

Reviewer #2 (Remarks to the Author):

I am happy with the response and have no objections to the paper being published

The authors kindly thank this reviewer for her/his support and positive feedback.

Reviewer #3 (Remarks to the Author):

The authors thoughtfully addressed all of my concerns.

The authors are delighted to have addressed all points of this reviewer and thank her/him for her effort and input.

****
